# Biocidal Inactivation of *Lactococcus lactis* Bacteriophages: Efficacy and Targets of Commonly Used Sanitizers

**DOI:** 10.3389/fmicb.2017.00107

**Published:** 2017-02-02

**Authors:** Stephen Hayes, James Murphy, Jennifer Mahony, Gabriele A. Lugli, Marco Ventura, Jean-Paul Noben, Charles M. A. P. Franz, Horst Neve, Arjen Nauta, Douwe Van Sinderen

**Affiliations:** ^1^School of Microbiology, University College CorkCork, Ireland; ^2^APC Microbiome Institute, University College CorkCork, Ireland; ^3^Laboratory of Probiogenomics, Department of Life Sciences, University of ParmaParma, Italy; ^4^Biomedical Research Institute, Hasselt UniversityDiepenbeek, Belgium; ^5^Department of Microbiology and Biotechnology, Max Rubner-InstitutKiel, Germany; ^6^FrieslandCampinaAmersfoort, Netherlands

**Keywords:** phage, fermentation, starter culture, dairy, resistance, chemical

## Abstract

*Lactococcus lactis* strains, being intensely used in the dairy industry, are particularly vulnerable to members of the so-called 936 group of phages. Sanitization and disinfection using purpose-made biocidal solutions is a critical step in controlling phage contamination in such dairy processing plants. The susceptibility of 36 936 group phages to biocidal treatments was examined using 14 biocides and commercially available sanitizers. The targets of a number of these biocides were investigated by means of electron microscopic and proteomic analyses. The results from this study highlight significant variations in phage resistance to biocides among 936 phages. Furthermore, rather than possessing resistance to specific biocides or biocide types, biocide-resistant phages tend to possess a broad tolerance to multiple classes of antimicrobial compounds.

## Introduction

Strains of *Lactococcus lactis* are among the most economically important lactic acid bacteria (LAB), being utilized in over two thirds of all commercial milk fermentations, and thus playing a vital role in the production of fermented products such as cheeses, buttermilk, and sour cream (Deveau et al., 2006). However, their widespread use is accompanied by the constant threat of (bacterio) phage attack and, despite continual research efforts into the prevention of phage infection, phage predation of lactococcal strains continues to be a problem. Infection by phages may result in lysis of the starter culture which interrupts the fermentation process, reduces the quality of the end-product, and may even result in complete fermentation failure (Garneau and Moineau, 2011). Contributing to this threat is the introduction of phages at various points in the fermentation process, such as (i) the intake of raw milk, in which phages may reside (Madera et al., 2004; Atamer et al., 2009); (ii) re-introduction of processed fermentation by-products, such as recycled whey protein; (iii) movement of employees between different areas of the facility; (iv) the spread of phages throughout the plant via aerosols (Verreault et al., 2011); and (v) ineffective sanitization of equipment between fermentations.

Significant technological and procedural advances have been made in an attempt to control phage contamination. These include (i) heat treatment of milk via pasteurization; (ii) high pressure treatments; (iii) the use of strain rotations and so-called direct vat starters (DVS) to prevent the proliferation of phages, along with the concomitant development of phage-resistant strains for use in these rotations (Moineau, 1999); (iv) the improvement of dairy plant facilities, such as plant design optimization and the use of closed vats (Allison and Klaenhammer, 1998); and (v) the utilization of commercial chemicals for the sanitization and disinfection of plant equipment and facilities. While these strategies have been relatively effective, with complete product loss now very rare (Madera et al., 2004), phage-associated fermentation issues are still a very common occurrence in dairy plants, probably because phages have adapted to overcome one or more of the imposed hurdles (Atamer et al., 2011; Mercanti et al., 2012; Murphy et al., 2014).

In dairy processing plants, sanitization between fermentations is a critical step in the control of phage contamination. This involves detailed cleaning in place (CIP) procedures, employing purpose-made chemical sanitizers for the physical and chemical removal of phages and other microbial contaminations (Cords et al., 2001). For biocides to be considered eligible for use in the dairy industry a number of criteria must be met, such as ease of use, cost effectiveness, lack of impact on the safety of workers and the final product and, of course, its anti-microbial efficiency (Guglielmotti et al., 2011). The application of food contact sanitizers is highly regulated (Wessels and Ingmer, 2013). For example, in Europe, sanitizers must have a demonstrated ability to reduce phage numbers by at least four logs under recommended test conditions before they can be deemed suitable for phage inactivation (European Committee for Standardization (CEN), 2002). Food contact sanitizers employ a range of active chemical agents, such as quaternary ammonium compounds, chlorine compounds, hydrogen peroxide, and iodine compounds (Gaulin et al., 2011), with many of these agents having been in use as disinfectants and preservatives for many decades or even centuries (McDonnell and Russell, 1999).

The precise mode of action of many antimicrobial compounds on bacteria has been widely studied, with much now known about the specific targets and anti-bacterial mechanisms of many biocides (McDonnell and Russell, 1999; Maillard, 2002; Wessels and Ingmer, 2013). In contrast, relatively scarce data currently exists pertaining to the virucidal mode of action of biocides (Garneau and Moineau, 2011; Murphy et al., 2014). However, while precise structural targets in phages are, as yet, largely uncharacterised, numerous studies have been performed on the efficacy of phage inactivation by commercially employed biocides. For example, a number of studies have been performed on the effectiveness of peracetic acid and sodium hypochlorite as virucidal agents (Binetti and Reinheimer, 2000; Capra et al., 2004; Avsaroglu et al., 2007). Quaternary ammonium compounds have also proved effective (Campagna et al., 2014), as has sodium hydroxide (Murphy et al., 2014). However, despite the proven effectiveness of these biocides, phages continue to persist in dairy facilities, and a possible contributing factor to this may be variations and/or increases in phage resistance to biocides.

The current study assessed the effectiveness of a range of commonly employed sanitizers in the neutralization of lactococcal phages of the industrially significant 936 group (Mahony et al., 2012; Murphy et al., 2016). This study represents the most in-depth investigation of dairy phage resistance to sanitizers to date, involving 36 bacteriophages and 14 anti-microbial compounds. Targets of these biocides were investigated through comparative proteomic analysis, mass spectrometry, and electron microscopy.

## Methods and materials

### Bacterial strains and bacteriophages

Thirty-five lactococcal 936-group bacteriophages previously isolated from whey derived from mixed starter systems in three Dutch cheese-producing plants (Murphy et al., 2013, 2016) were examined in this study, along with an additional phage (i0139), which had also been isolated at a later date from one of these whey samples. The bacterial host strains employed to propagate these phages are listed in Table 1. Bacterial strains were cultured overnight at 30°C in M17 broth (Oxoid, Hampshire, UK) supplemented with 0.5% w/v lactose (LM17). Phages were propagated in 10 ml of this medium, supplemented with 10 mM CaCl_2_, then filtered through a 0.45 μm filter (Sartorius, Dublin, Ireland), and stored at 4°C.

**Table 1:** **Phages and host strains used in this study**.

**Phage**	***L. lactis* host**	**Phage references**	**Phage**	***L. lactis* host**	**Phage references**
A.16	A	Murphy et al., 2016	145	M	Murphy et al., 2013
19	A	Murphy et al., 2013	109	M	Murphy et al., 2013
4	A	Murphy et al., 2013	M.5	M	Murphy et al., 2016
A1127	A	Murphy et al., 2016	93	M	Murphy et al., 2013
Lj	A	Murphy et al., 2013	M.16	M	Murphy et al., 2016
C0139	C	Murphy et al., 2016	M1127	M	Murphy et al., 2016
D.18	D	Murphy et al., 2016	155	M	Murphy et al., 2013
43^*^	D	Murphy et al., 2013	4.2	4	Murphy et al., 2016
17	E	Murphy et al., 2013	5.12^*^	5	Murphy et al., 2016
E1127	E	Murphy et al., 2016	91127	9	Murphy et al., 2016
F0139^*^	F	Murphy et al., 2016	16	9	Murphy et al., 2013
F.17	F	Murphy et al., 2016	10.5	10	Murphy et al., 2016
G^*^	G	Murphy et al., 2013	114	11	Murphy et al., 2013
i0139^*^	i	This study	44	11	Murphy et al., 2013
L.18	L	Murphy et al., 2016	13.16	13	Murphy et al., 2016
L.6	L	Murphy et al., 2016	15	13	Murphy et al., 2013
40	L	Murphy et al., 2013	19.2	19	Murphy et al., 2016
129	M	Murphy et al., 2013	19.3	19	Murphy et al., 2016

All phages are members of the 936-group. All host strains are as described by Murphy et al. (2013). All phages were isolated against these strains over a period of 4 years (Murphy et al., 2013, 2016). Phages denoted by

* *are those which exhibited resistance to more than one biocide during assays*.

### Biocides

A range of biocidal compounds that are commonly employed in the food and beverage industry or that were traditionally used as biocidal agents were investigated for their phage-inactivating capabilities. A list of the compounds and solutions assessed in this study are itemized in Table 2. These were purchased either as pure powders/solutions or as mixtures of compounds in the case where purpose-made sanitizers designed for the dairy industry were employed. As sodium hypochlorite and peracetic acid have recently been examined using a number of the phages involved in this study (Murphy et al., 2014), these were omitted.

**Table 2:** **Biocidal agents investigated during this study**.

**Compound/Solution**			**Concentrations (%)**
**PURE COMPOUNDS/SOLUTIONS**			
Benzalkonium chloride^*^			0.08
Polyvinylpyrrolidone-iodine^*^			1
Hydrogen peroxide^*^			14
Ethanol			40, 80
Isopropanol			40, 80
Sodium percarbonate			1, 5, 10
Sodium dichloroisocyanurate			5, 10, 20
Sodium chlorite			1, 5, 10, 20
**Industrial sanitizer solutions**	**Composition**	**Manufacturer recommended concentrations (%)**	
Sanitizer A^*^	C8-C18 alkyldimethyl chloride ammonium compound	1–5	0.08
Sanitizer B^*^	Mixture containing 30–60% sodium hydroxide (<0.06% at concentration employed)	0.5–2	0.1
Sanitizer C^*^	Mixture containing 5–10% sodium hydroxide (<0.012% at dilution), 2–5% sodium hypochlorite (<60 ppm at dilution)	1	0.12
Sanitizer D	Ethanol 10%, chlorhexidine digluconate 10%, Tetradecyl-trimethyl-ammonium-bromide <1%	12–20	16, 40, 80
Sanitizer E^**^	Polymeric-biguanide-hydrochloride	2–5	0.1, 1, 5, 10
Sanitizer F^**^	Mixture containing 30% nitric acid, 5% orthophosphoric acid	1	0.1, 0.5, 1, 5

Summary of the biocides employed in this study. All pure compounds/solutions were purchased from Sigma Aldrich (Wicklow, Ireland). Biocides marked by

* were selected for in depth study due to the consistent and effective results produced. Biocides marked by

** *produced inconsistent results, preventing further study. All other biocides proved ineffective for the purpose of phage elimination at the concentrations examined*.

### Biocide exposure protocol

The biocide exposure protocol was adapted from Neve et al. (1996), which formed the basis for the European standard EN 13610:2002. Briefly, 10 μl of phage lysate was diluted in 90 μl of water, and then mixed with 100 μl of 10% milk whey to simulate conditions in dairy facilities. This typically produced a starting phage titer of 10^7^ plaque-forming units (PFU) ml^−1^. The lysate and whey were mixed for 2 min, and 800 μl of biocide at the appropriate concentration(s) was then added. Samples were taken immediately prior to biocide addition (t_0_) and at 2, 10, 20, and 30 min following the addition of the particular biocide. At each of these time-points, 20 μl of the mixture was removed and immediately placed in 980 μl neutralization buffer (M17 supplemented with 3% Tween 80, 3% sodium thiosulfate, 0.3% L-cysteine, and 0.3% L-histidine) to neutralize chemical activity (Campagna et al., 2014). The phage titer at each time-point was determined via the double-layer plaque assay method (Lillehaug, 1997). The biocide concentration to be used was determined by first testing each biocide against three phages (named 93, G, and 4.2) selected as representatives of the 36 phages included in this study. These three phages were tested in optimization trials against a range of concentrations for each chemical in order to first determine the lethal concentration, i.e., the concentration required to cause a complete elimination of detectable phages within 30 min. From this lethal concentration, a sub-lethal concentration was determined, which was defined as the concentration at which at least a 3-log reduction in efficiency of plaquing (E.O.P.) was observed for the majority of phages. This concentration was selected to test against the full set of phages in order to observe variations in resistance levels. Resistant phages were defined as those which suffered less than a three log reduction in detectable phages after 30 min of exposure to a particular biocide. Intermediate resistance was defined as a decrease in surviving phages of three to five logs. For each phage assay, a control was performed by adding distilled water in place of sanitizer. Controls were also performed both with and without neutralization buffer, and in the presence and absence of milk whey, in order to validate their expected impact on phage titer and stability.

### Effect of biocides on the phage proteome

In order to determine possible targets of biocidal action, SDS-PAGE analysis of phage proteins following biocide exposure was performed. This was assessed by comparing the protein profiles of purified phages before and after biocide exposure. Phages were first concentrated and purified by a discontinuous cesium chloride density gradient. Fifty micro liter of purified phage (representing approximately 10^11^ PFU ml^−1^) was then mixed with 150 μl of the relevant chemical compound. After 30 min, 200 μl of neutralization buffer was added to halt biocidal activity as described above. Phage protein material was concentrated by chloroform/methanol precipitation as performed by Casey et al. (2014). The sample was applied to a 12% SDS-PAGE gel, and the resulting protein profiles were visualized following staining with 0.25% Coomassie Brilliant Blue (Bio-Rad, Hertfordshire, UK). The SDS-PAGE-generated protein profiles of phages following chemical exposure were then compared with controls in order to identify proteins that had been targeted by biocidal activity. Controls consisted of purified phage mixed with distilled water both in the presence and absence of neutralization buffer to ensure the latter had no effect on the protein profile. In addition to Coomassie Blue staining, silver staining (Chevallet et al., 2006) was performed to obtain a higher resolution of possible differences in phage proteomes before and after chemical exposure.

Western blot analysis was performed on a number of phage proteins, namely the major tail protein (MTP) of phage 93, the tail protein extension (TpeX) of phage C0139, and the receptor binding protein (RBP) of phage 4.2, using antibodies described in a previous study by Murphy et al. (2016). Phages C0139 and 4.2 were used since antibodies proved specific to these phages. Additionally, these phages served as controls to ensure that effects observed in phage 93 were not unique to this phage, and were representative of other 936 phages. Western blot analysis was performed as described previously (Collins et al., 2013).

### Mass spectrometry

In order to identify proteins affected by biocidal agents employed in this study, mass spectrometry analysis was performed on a representative phage, i.e., phage 93. Protein bands derived from SDS-PAGE analysis were excised, digested with trypsin, and analyzed by electrospray ionization-tandem mass spectrometry (ESI-MSI/MS) as performed previously (Ceyssens et al., 2008; Holtappels et al., 2015). Phage-associated protein bands that newly appeared following exposure to biocides, thereby indicating damage to specific structural proteins of the phage, were also analyzed using the same approach in order to identify the affected protein.

### Electron microscopy imaging of the effects of biocides on phage structure

Phage 93 was first concentrated by precipitation with polyethylene glycol 8000 (PEG_8000_) (10% final concentration) and purified using two CsCl density gradients; the first gradient at 82,000 × g for 2.5 h, the second at 340,000 × g for 18 h, as described by Marcó et al. (2012). For visualization of the effects of biocides on virion integrity, 50 μl of purified phage particles (representing ~10^11^ PFU ml^−1^) was mixed with 150 μl of biocide for 30 min, followed by the addition of 200 μl of neutralization buffer. Staining was performed with 2% (w/v) uranyl acetate on freshly prepared carbon films. Grids were analyzed in a Tecnai 10 transmission electron microscope (FEI Thermo Fisher Scientific, Eindhoven, The Netherlands) at an acceleration voltage of 80 kV. Micrographs were taken with a MegaView G2 charge-coupled device camera (Emsis, Münster, Germany; Vegge et al., 2006).

### Sequencing of phage i0139

Phage i0139 DNA was isolated as described by Murphy et al. (2013). Genomic libraries were generated using 2.5 μg of genomic DNA and were fragmented with Bioruptor NGS ultrasonicator (Diagenode, USA) followed by size evaluation using Tape Station 2200 (Agilent Technologies). A whole fragmented library was constructed using the TruSeq DNA PCR-Free LT Kit (Illumina). Samples were loaded into a Flow Cell V3 600 cycles (Illumina) as reported by technical support guide. Reads were depleted of adapters, quality filtered and assembled through using MIRA v4.0.2. Open reading frame prediction was performed with Prodigal v2.6, and the quality of the final contigs was improved with Burrows-Wheeler Aligner, SAMtools suite, and VarScan v2.2.3. Annotation of the ORFs was performed using the MEGAnnotator pipeline (Lugli et al., 2016). The annotated genome was deposited in Genbank under accession number KX379665.

### Phylogenetic analysis

Phylogenetic analysis was performed on all phages in order to investigate a possible genetic basis for biocide resistance. Whole genome nucleotide sequences, the concatenated amino acid sequence of all coding sequences (CDS), and the amino acid sequences of individual structural proteins were all compared. Whole phage genome nucleotide alignments were performed using clustalW (Thompson et al., 1994). The phylogenetic tree was computed by the maximum-likelihood method in PhyML v3.0 and bootstrapped x1000 replicates. The final tree file(s) was visualized using ITOL (Interactive Tree of Life; http://itol.embl.de/index.shtml). The amino acid sequences of all coding regions were concatenated and aligned in MEGA6 using MUSCLE. Trees were constructed using the maximum likelihood method (Enright et al., 2002). Amino acid sequences of individual proteins were compared using a similar approach.

## Results

### Phage inactivation assays with pure compounds

**Figure 1:**
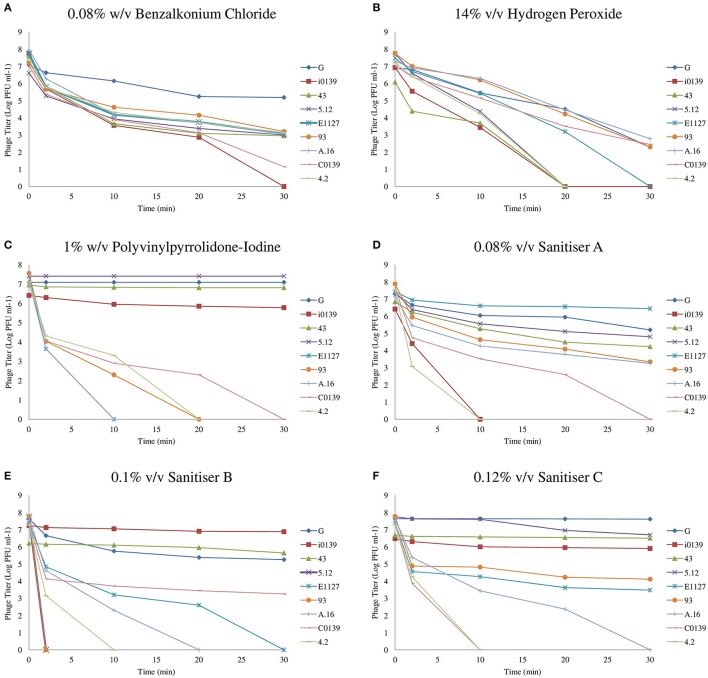
**Resistance profiles of a selection of nine representative phages from the 36 examined. (A)** Phage Inactivation kinetics at 0.08% w/v benzalkonium chloride. **(B)** Phage inactivation kinetics at 14% v/v hydrogen peroxide. **(C)** Phage Inactivation kinetics at 1% w/v PVP. **(D)** Phage inactivation kinetics at 0.08% v/v Sanitiser A. **(E)** Phage inactivation kinetics at 0.1% v/v Sanitiser B. **(F)** Phage inactivation kinetics at 0.12% v/v Sanitiser C. All graphs represent the average of at least triplicate assays.

An overview of the results produced by biocide exposures can be found in Figure 1, where the effect of biocides on the titer of a selection of nine representative phages has been outlined. These nine representatives include resistant, intermediately resistant, and susceptible phages, and were selected to best represent the diversity and range of biocide resistance observed.

Benzalkonium chloride (BAC), a quaternary ammonium compound (QAC), was the most effective pure compound examined, resulting in a complete elimination of detectable phages at a concentration of just 0.1% w/v after 30 min (data not shown). The sub-lethal concentration chosen for this compound was 0.08% w/v, which caused a reduction in phage titer of at least four logs for the majority of phages (Supplementary Table S1). Little variance in resistance between phages was observed upon exposure to BAC (Figure 1A), with G being the only phage exhibiting substantial resistance, suffering less than a two log reduction after 30 min exposure time.

Hydrogen peroxide proved a less effective virucidal agent and required a high concentration of 20% v/v to cause complete phage inactivation, while 14% v/v was chosen as a suitable sub-lethal concentration. Similar to BAC, little variation in phage resistance was observed upon exposure to this chemical. A number of phages demonstrated a slightly higher susceptibility, while the majority of phages suffered a 3–5 log reduction after 30 min (Figure 1B, Supplementary Table S2).

Polyvinylpyrrolidone-iodine (PVP) is an iodophor complex containing approximately 9–12% available iodine in soluble powder form. A concentration of 4% w/v PVP was required to achieve complete inactivation of all representative phages. Phage G was observed to be resistant to 3% w/v PVP with less than a single log reduction (data not shown). However, all other examined phages were eliminated within 20 min, and thus a sub-lethal concentration of 1% w/v was established. At this concentration, seven resistant phages were identified: G, 5.12, 43, D.18, F.17, F0139, and i0139. Phages exhibited a wide spectrum of susceptibility to PVP, with a number of phages completely inactivated after 10 min, while other phages exhibited less than a single log reduction after 30 min (Figure 1C, Supplementary Table S3).

Ethanol, isopropanol, sodium percarbonate, sodium chlorite, and sodium dichloroisocyanurate were ineffective virucidal agents against this phage collection, with less than a single log reduction observed in the representative phages at all examined concentrations after 30 min (data not shown).

### Industrial sanitizer solutions

In addition to assessing the impact of a variety of pure compounds, this study also aimed to assess the effect of commercially available industrial sanitizers, many of which are composites of two or more compounds. Sanitizer A is a QAC-based disinfectant designed for surface cleaning in the food and dairy industry. It was observed to be the most effective of the commercial sanitizers examined with high efficacy concentrations below the supplier's recommended dilution and with an observed phage-elimination concentration of 0.1% v/v. A sub-lethal concentration of 0.08% v/v was established, to which four phages (G, 43, 5.12, and E1127) were shown to be resistant. Considerable variation in resistance was observed among phages with this sanitizer solution (Figure 1D). For example, a large group of phages (16 in total) exhibited intermediate biocide tolerance, i.e., causing a three to five log reduction in plaquing efficiency (Supplementary Table S4), while other phages, such as 4.2, exhibited high susceptibility (Figure 1D).

Sanitizer B is an alkaline detergent mixture, with sodium hydroxide as its main active ingredient. This biocide was observed to provide a powerful effect in reducing phage infectivity at low concentrations, with a concentration of 0.14% v/v sufficient for the elimination of 10^7^ PFU ml^−1^ phages. Again, this is significantly lower than the recommended dilution of 0.5–2%. At a concentration of 0.1% v/v, large variations in phage survival were observed (Figure 1E). Four resistant phages were identified (G, i0139, 43, and F0139), and five phages exhibited intermediate resistance, while infectivity of five phages was undetectable after just two min of exposure (Supplementary Table S5).

Sanitizer C was shown to eliminate all detectable phages at a concentration of 0.15%, once again far below manufacturer recommendations, and variations in phage resistance were observed at a concentration of 0.12% v/v (Figure 1F), with five resistant phages identified: G, i0139, 43, F0139, and 5.12. The majority of phages exhibited an intermediate resistance at this concentration, while infectivity of three phages (C0139, 4.2, and 13.16) was undetectable after 10 min of exposure (Supplementary Table S6).

Sanitizer D, a quaternary ammonium compound-based sanitizer, was ineffective at the concentrations examined (data not shown). Sanitizers E and F produced inconsistent results at all concentrations examined, with phage titers appearing to rise and fall between time-points and dilutions (data not shown), thus indicating that these sanitizers may cause phage particle aggregation. However, while the results were inconsistent and thus preventing further analysis of these sanitizers, it was clear that significant levels of phages survived exposures at concentrations above those recommended by the manufacturers.

As can be seen from these results, phages exhibiting resistance to one biocide generally appear to be resistant to multiple sanitizing agents. Phage G exhibited resistance to all biocides other than hydrogen peroxide at the concentrations examined, while i0139, 43, F0139, and 5.12 all demonstrated resistance to at least three biocides. Phages D.18 and F.17 (only resistant to PVP) and E1127 (resistant to Sanitizer A alone) were found to be the only exceptions to this general observation.

### Effect of biocides on the phage proteome

**Figure 2:**
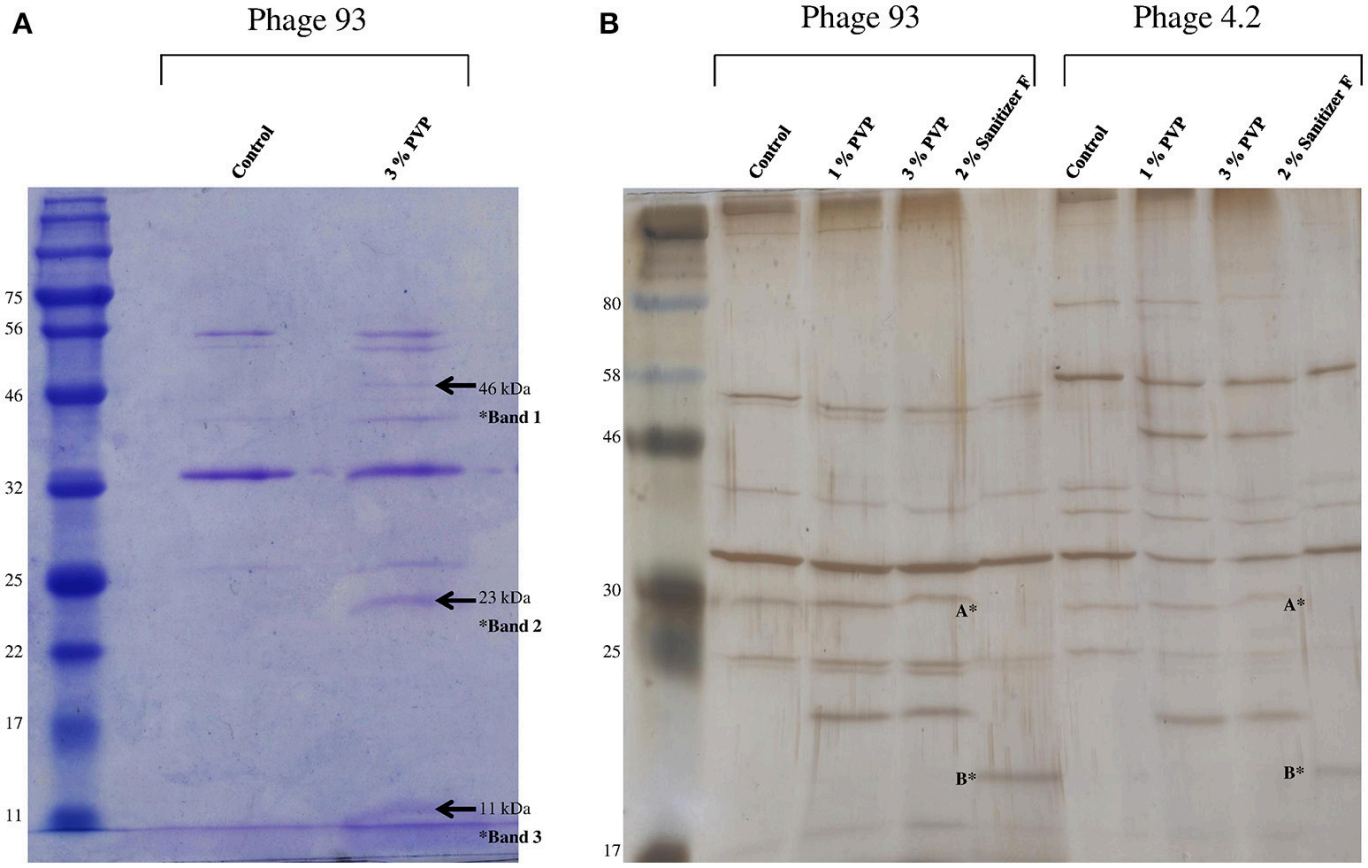
**(A)** 12% SDS-PAGE of purified phage 93 before and after exposure to 3% w/v PVP. The ladder used is the 11–190 kDa broad-range protein ladder (New England BioLabs). New bands emerging after PVP exposure are indicated by the arrows. **(B)** Silver stain analysis of phages 93 and 4.2 before and after exposure to 1% PVP, 3% PVP, and 2% v/v Sanitizer F. Apparent is the disappearance of a band of 30 kDa (indicated by A^*^) after exposure to Sanitizer F in both phages, with the appearance of a new band of 20 kDa (indicated by B^*^). The ladder used is the 7–175 kDa broad-range protein ladder (New England BioLabs).

Phage 93 was selected as a representative phage for the analysis of the effects of biocides on phage structural elements as it demonstrated susceptibility to all biocides in addition to being a strong propagator. Comparison of the phage structural protein profile before and after biocide exposure via Coomassie staining revealed the appearance of two additional bands corresponding to approximately 23 kDa and 11 kDa, as well as a less prominent band estimated at 46 kDa, upon exposure to PVP, indicating damage of structural phage components (Figure 2A). Silver stained PAA gels also revealed the appearance of additional bands following PVP treatment in phage 4.2 (Figure 2B), and other phages (data not shown), demonstrating that this damage was not unique to phage 93. Silver stains also revealed the disappearance of a band of 30 kDa and the emergence of an additional band of 20 kDa following exposure to Sanitizer F, which caused phage aggregation during biocide exposure assays. However, this unfortunately could not be reproduced by the less sensitive Coomassie staining. Protein profiles following exposure to sanitizers B and C were impossible to visualize, possibly due to the highly basic nature of sodium hydroxide. Exposure to Sanitizer A, benzalkonium chloride, and Sanitizer E did not caused any obvious change in protein profile (data not shown).

**Figure 3:**
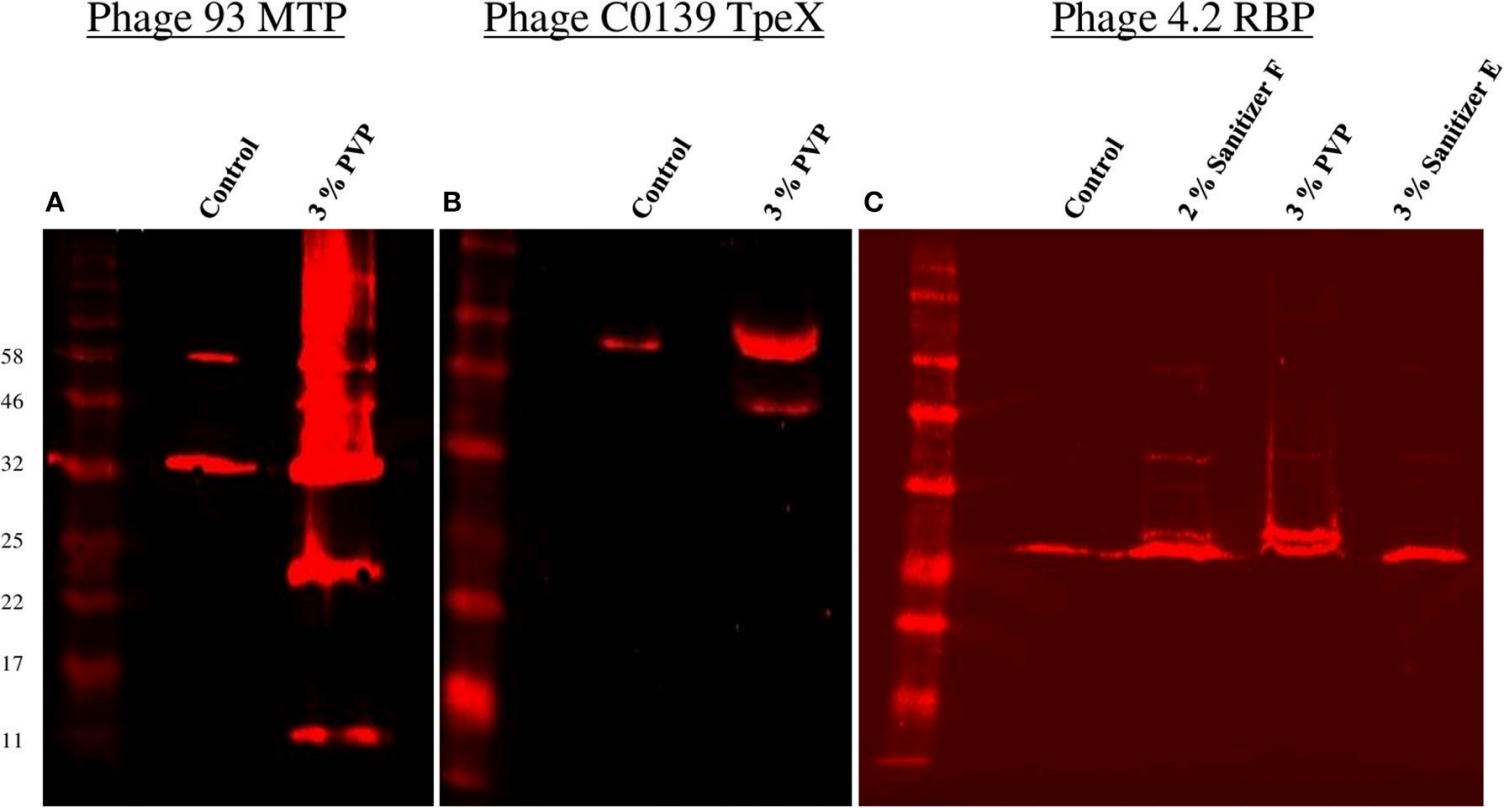
**Western hybridisation analysis of the effects of a selection of biocides on a number of phage proteins. (A)** Western hybridisation analysis of phage 93 using anti-MTP antibodies. **(B)** Western hybridisation analysis of phage C0139 using anti-TpeX antibodies. **(C)** Western hybridisation analysis of phage 4.2 using anti-RBP antibodies. For all blots, the New England Biolabs Color Prestained Protein Standard, Broad Range (11–245 kDa) was used.

Thus, PVP proved to be the only biocide which produced consistent visible alterations to the phage protein profile. In order to possibly identify the proteins in which damage was observed, Western hybridisation analysis was performed on the phage profile after PVP exposure using anti-MTP (Figure 3A) and anti-TpeX (Figure 3B) antibodies for phages 93 and C0139, respectively. The obtained results indicate that the protein bands emanating from the treatment process were composed of the major tail protein (MTP), with the 46 kDa band of containing the tail protein extension (TpeX), which is attached to the C-terminus of a proportion of MTPs in the mature phage (Murphy et al., 2016). These results demonstrate that PVP causes very specific degradation of the MTP protein, resulting in two products: a smaller N-terminal portion (11 kDa), and a larger C-terminal protein fragment (23 kDa), with a proportion of the latter protein fragment also possessing the attached TpeX protein (46 kDa).

Western hybridisation analysis was also performed using anti-RBP antibodies for phage 4.2 (Figure 3C). Visible alterations in the protein profile were apparent after exposure to two biocides, PVP and Sanitizer F, with little change observed upon exposure to Sanitizer E. In the case of PVP and Sanitizer F, an additional band was visible directly above the protein band corresponding to the native RBP, with a number of faint larger bands also visible, indicating that these biocides affect the structural integrity of the RBP.

The untreated phage proteomic profile, in addition to the emergent bands caused by the addition of PVP, was analyzed via electrospray ionization-tandem mass spectrometry (ESI-MS/MS; Table 3). Most of the predicted tail-associated proteins of phage 93, such as the major tail protein, the tape measure protein, the TpeX protein, and the receptor binding protein, were detected in the analysis of the control profile, although phage capsid proteins were not identified. Analysis of the bands that are formed following PVP exposure confirmed the composition of these bands as sections of the MTP and TpeX. From the peptide reads obtained from each band, it was also possible to narrow the location of PVP damage in the MTP to within 13 amino acid residues (Supplementary Figure S1). In the 11 kDa band, a number of smaller tail-associated proteins (hypothetical structural proteins 1, 2, 3, and 4) were detected which had not been identified in the control profile, as well as fragments of the portal protein, located at the base of the capsid (Wikoff et al., 2000), and the protease protein, involved in capsid assembly (Cheng et al., 2004). The identification of peptides that originate from the portal protein in the smallest band of 11 kDa hints at further structural damage to the tail resulting from PVP exposure.

**Table 3:** **Summary of mass spectrometry data**.

**Phage**	**ORF**	**Putative function**	**No. of peptides**	**Coverage (%)**
**93 (FIGURE 2A. CONTROL)**				
	07	Portal protein	12	38
	15	Major tail protein	10	51
	16	Tail protein extension	6	53
	19	TMP	19	24
	20	Dit	14	33
	21	Tal	8	24
	23	RBP	11	58
**93 PVP (FIGURE 2A 3% PVP)**				
**Band 1:**	16	Tail protein extension	4	44
	15	Major tail protein	3	12
**Band 2:**	15	Major tail protein	4	20
**Band 3:**	16	Tail protein extension	2	17
	16	Major tail protein	4	29
	7	Portal protein	3	10
	8	Protease	6	48
	10	Hypothetical structural protein 1	3	44
	11	Hypothetical structural protein 2	2	27
	13	Hypothetical structural protein 3	1	14
	14	Hypothetical structural protein 4	4	35

*Structural proteins extracted from purified phage 93 particles before (93) and after (93 PVP) exposure to 3% w/v PVP via electrospray ionization-tandem mass spectrometry (ESI-MSI/MS). A minimum of either two independent unique peptides or 5% sequence coverage were used as threshold values*.

### Impact of biocides on the phage structure and integrity

**Figure 4:**
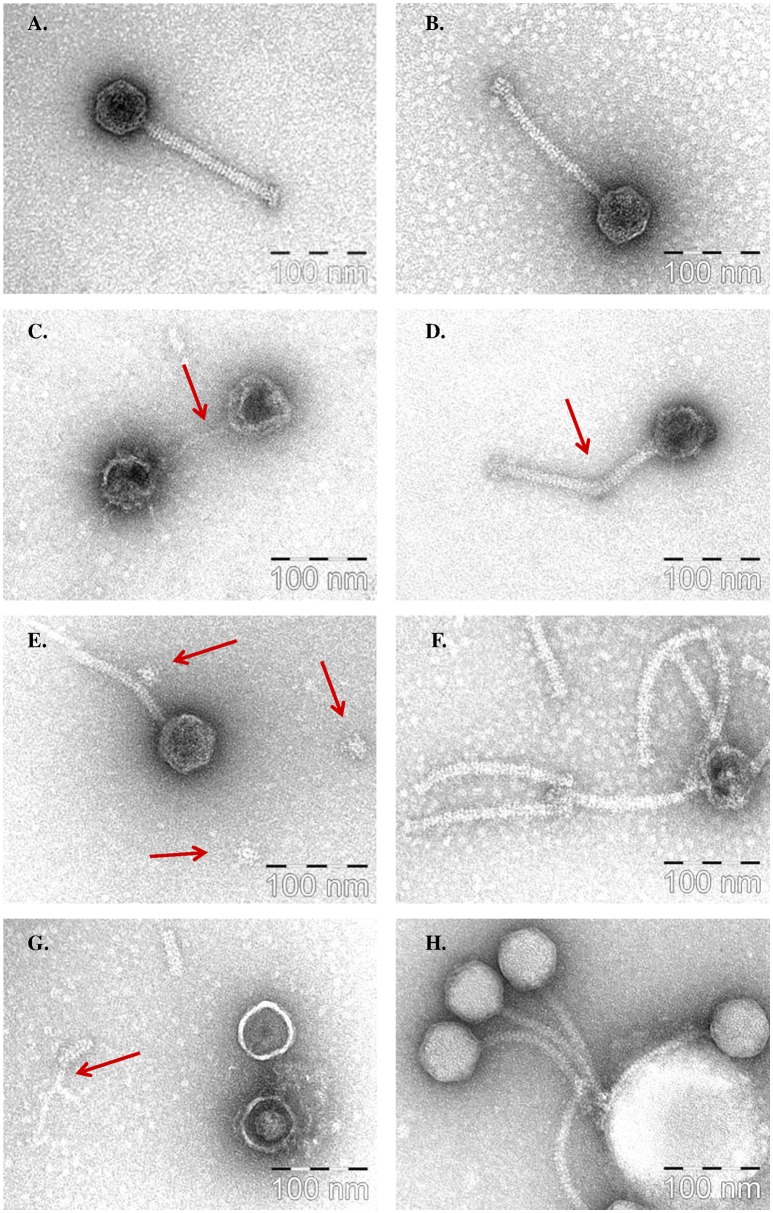
**Representative electron microscopy images of the effects of four biocides on the structure of phage 93. (A)** Control. **(B)** Neutralisation buffer control. **(C)** 3% PVP, with possible phage DNA indicated by the red arrow. **(D)** 1% PVP with break-point of tail indicated by the red arrow. **(E)** 1% PVP with possible detached baseplates indicated by the red arrow. **(F)** 0.5% Sanitizer A. **(G)** 0.5% Sanitizer B, with possible tape measure protein (TMP) remaining after the degradation of the major tail protein (MTP) indicated by the red arrow. **(H)** 3% Sanitizer D.

Electron microscopic imaging was performed on phages following exposure to a selection of biocides in an attempt to further elucidate structural targets of biocidal action. The four biocides selected were: PVP; sanitizers A and D (which demonstrated no visible damage to the structural proteome); and Sanitizer B (a sodium hydroxide-based agent that could not be subjected to proteomic analysis). Analysis of the effects of exposure was performed on the representative phage 93. Resulting images demonstrated that each compound induced unique effects on the phage structure, with each appearing to have distinct primary structural targets in the phage (Figure 4).

In the case of PVP, phage 93 was first imaged after exposure to a 3% w/v solution, as this concentration produced the clearest differentiating effect in protein profiling assays. However, for electron microscopic imaging, this appeared to be too potent, with phage tails completely destroyed and the phage head appearing collapsed and empty, with possible remaining DNA strands extending from the capsid (Figure 4C, Supplementary Figure S2A). Thus, a range of concentrations between 1 and 2% were examined in order to image the first signs of critical damage. A concentration of 1.9% PVP proved to produce the clearest signs of structural damage. Consistently, the tail appeared to be the primary target, with an obvious break in the tail observed (Figure 4D, Supplementary Figure S2B). Furthermore, multiple occurrences of possible detached baseplates, a structure located at the end of the phage tail responsible for host recognition and binding (Mahony and van Sinderen, 2012), were observed, in parallel with the absence of baseplates in a number of phage tails (Figure 4E, Supplementary Figure S2C). Damage to the phage capsid of a similar but less extreme nature to that witnessed at 3% was also observed at this concentration, although it was not as prevalent or comprehensive as the observed damage to the tail.

Sanitizer A (QAC) was examined at two concentrations: 0.5 and 1% v/v (which are the supplier's recommended CIP dilutions for food industry). At 0.5%, the main effects observed were damaged, empty capsids, and detached but intact phage tails (Figure 4F, Supplementary Figure S2D). At 1%, capsids appeared even further damaged, while tails were again detached but intact (Supplementary Figure S2E). Thus, these images suggest the phage capsid to be one of the main targets of Sanitizer A.

Sanitizer B was similarly examined at 0.5 and 1% v/v, concentrations within the manufacturer's recommended usage dilutions. At 1%, phage tail structures appeared mostly destroyed, with just isolated fragments of the tail remaining, while the capsid suffered significant damage, appearing empty and collapsed (Supplementary Figure S2G). At 0.5%, a clearer image was obtained (Figure 4G, Supplementary Figure S2F). Significant damage to the phage capsid structures was again observed, with capsids appearing shrunken and much “smoother” than the normal icosahedral shape, while many fragments of detached phage tails were present, with possible sections of tail tubes visible where the surrounding MTP had been damaged.

Sanitizer E was examined at concentrations of 1 and 3% v/v. At 1%, phages appeared to be largely structurally intact (Supplementary Figure S2H). However, in a number of instances small white objects, thought to be biocide residues, were attached to the phage capsid. At 3%, phages still appeared to be structurally intact (Figure 4H, Supplementary Figure S2I). However, at this concentration biocide residues were much larger, and phages displayed a clear tendency to attach to and cluster around these residues. Additionally, many phage capsids appeared enlarged and much brighter than the control, indicating that the biocide is attaching to, or possibly even entering the phage capsid. These images suggest that, rather than destroying the phage, Sanitizer E binds to the phage and causes the phages to aggregate.

### Comparative genomic analysis

**Figure 5:**
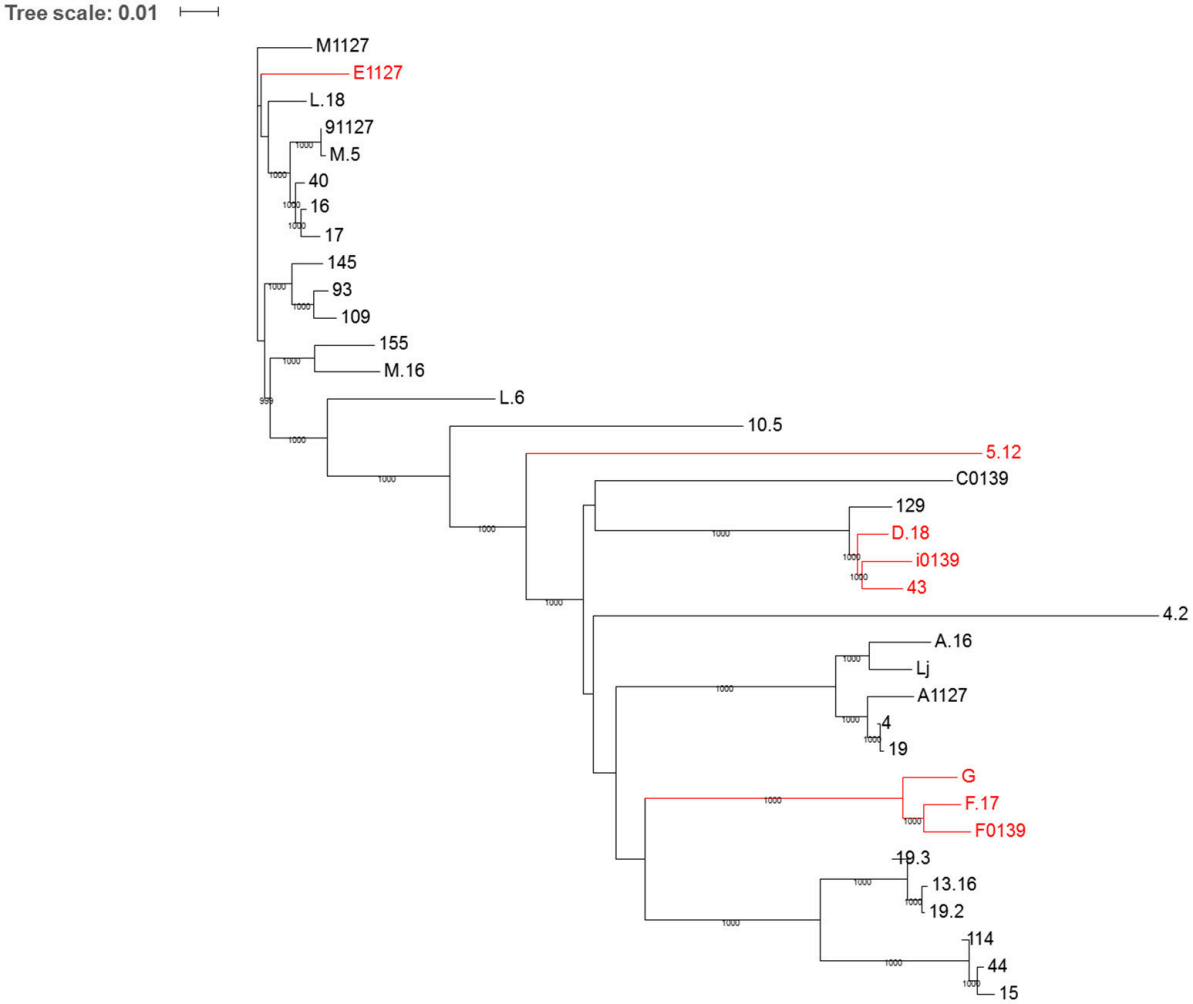
**Phylogenetic tree of the 36 bacteriophages used in this study based on whole genome nucleotide sequence**. Phages demonstrating resistance to at least one biocide are highlighted in red. Whole phage genome nucleotide alignments were performed using clustalW. The phylogenetic tree was computed by the maximum-likelihood method in PhyML v3.0 and bootstrapped x1000 replicates. The final tree file was visualized using ITOL (Interactive Tree of Life; http://itol.embl.de/index.shtml).

Phage sequences were compared at both the nucleotide and amino acid levels via the construction of phylogenetic trees in order to discern possible genetic differences between susceptible and resistance phages. Figure 5 represents a phylogenetic tree based on the whole genome nucleotide sequences of the phages. This analysis highlights that the majority (six out of eight) of phages exhibiting resistance to at least one biocide separate into two clades, while phage 5.12 separates into a distinct clade. Trees were also assembled based on the concatenated amino acid sequence of the overall predicted proteome of each phage, as well as the amino acid sequences of individual genes of the late region of the genome, which primarily encodes proteins involved in phage packaging, morphogenesis, and cell lysis (Chandry et al., 1997; Crutz-Le Coq et al., 2002). In all cases similar results to the nucleotide sequence tree were produced (data not shown), with six resistant phages separating into two clades, and phage 5.12 being clustered as a separate member from all other phages. Additionally, upon manual evaluation of nucleotide and amino acid sequence alignments of structural proteins, phages appeared to separate due to a range of nucleotide and amino acid substitutions throughout the sequence (data not shown). Thus, it appears that the separation of resistant from susceptible phages cannot be narrowed down to a specific mutation that affects a single protein or region of a protein, but, rather, is the result of an accumulation of multiple genetic adaptations throughout the whole genome.

## Discussion

This study aimed to assess the effect of a range of biocides typically employed in the dairy industry against a set of 36 lactococcal 936 group phages. The results of this study demonstrate that significant variations in resistance to biocidal action exist among members of this important phage group.

Quaternary ammonium compounds (QACs) have long been used as disinfectants (McDonnell and Pretzer, 2001). In this study, QAC-based biocides were found to be the most effective disinfectants of both the pure compounds and commercial mixtures examined, in congruence with previous studies (Campagna et al., 2014). However, their effectiveness was qualified in our study by the detection of at least one resistant phage for each QAC, while the ineffectiveness of Sanitizer D demonstrates that not all QAC-based biocides are suited for dairy phage inactivation. Furthermore, there is growing concern regarding QAC residues in the milk and dairy production chain (Danaher and Jordan, 2013). Thus, whilst they are highly effective as virucidal agents, QAC-based sanitizers may not be best suited to dairy processing environments.

PVP-iodine and other iodophor compounds are widely employed in the brewing industry and dairy farms (Castro et al., 2012). However, the 4% w/v PVP-iodine (4000 ppm available iodine) required to eliminate all phage particles greatly exceeds concentrations advised for use in the food industry. Additionally, the detection of the highest number of resistant phages of all examined biocides also supports the conclusion that iodine-based compounds are not suitable sanitizers for dairy facilities.

The effectiveness of sodium hydroxide against many of the phages utilized in this study has recently been investigated (Murphy et al., 2014), where 0.2% sodium hydroxide was observed to completely eliminate the assessed phages. In our study, two sodium hydroxide-based sanitizers (Sanitizers B & C) specifically designed for the dairy industry supported previous findings of the efficacy of sodium hydroxide as a virucidal agent. Since these are complex mixtures of chemicals, it cannot be precluded that a strong synergistic effect occurs between sodium hydroxide and sodium hypochlorite in Sanitizer C (and possibly between sodium hydroxide and the numerous unlisted components in Sanitizer B). While the use of either of these agents alone at the concentrations employed during assays would probably have little effect, their combined action results in highly effective phage inactivation.

The bactericidal and bacteriostatic properties of hydrogen peroxide are well established (Repine et al., 1981; Alasri et al., 1992; Pericone et al., 2000). However, it appeared highly unsuitable for the elimination of phages in this study. A number of other examined biocides proved even less effective, and in the case of ethanol and isopropanol their poor virucidal activity against dairy phages corroborates the results of Campagna et al. (2014), as well as van Engelenburg et al. (2002), who demonstrated the ineffectiveness of alcohol against non-lipid enveloped viruses.

The commercial Sanitizers E and F caused phage particle clumping and aggregation. In the case of Sanitizer E, a polymeric biguanide, this observation has previously been reported for the *Escherichia coli* phage MS2 (Pinto et al., 2010). While this phenomenon has not been studied in phages related to the dairy industry, aggregation has been reported to decrease the potency of antimicrobials against the non-enveloped poliovirus (Salk and Gori, 1960) and Norwalk virus (Keswick et al., 1985). Indeed, imaging confirmed the hypothesis that exposure results in phage aggregation rather than inactivation, and it appears that the biocide may be binding to, or entering the capsid structure. However, this does not appear to cause measurable inactivation of phages.

With the exception of hydrogen peroxide, at least one resistant phage was found for each biocide assayed in full, while inactivation times and surviving titers also proved to be highly diverse among the other phages. The fact that phages exhibiting resistance to one biocide tend to be resistant to others indicates that biocide resistance is, in part, not based on resistance to the mechanism of action of a specific biocide, but rather due to the intrinsic robustness of the phage itself. This hypothesis was further supported by phylogenetic analysis of both the whole genome nucleotide sequence and individual CDS, with phylogenetic trees consistently demonstrating separation between resistant and non-resistant phages. The fact that resistant phages continually separated at both the whole genome and individual CDS level again indicates that resistance is due to the cumulative effect of many genetic adaptations located throughout the phage genome.

While these data add significantly to previous studies on the efficacy of biocide treatments against dairy phages, there remains a distinct lack of understanding of the underpinning mechanisms of inactivation of bacteriophages by biocides. In this study, we have attempted to elucidate the structural targets of a number of biocides through a range of techniques.

Initially, the protein profile of phage 93 was compared before and after exposure to most of the biocides examined via SDS-PAGE. The most definitive change in the phage structural protein profile was observed after exposure to PVP, which produced additional bands confirmed to be composed of sections of the major tail protein via mass spectrometry. This is one of the first instances of a structural target of biocidal action being positively identified in a bacteriophage. Phage capsid proteins were not detected in the control possibly due to solubility, with a substantial insoluble fraction remaining after phage protein concentration potentially containing capsid components. This phenomenon has previously been observed in the temperate lactococcal phage r1t (van Sinderen et al., 1996), and *E. coli* phage HK97, where the covalent cross-linking of head components resulted in oligomers too large to enter the gel (Popa et al., 1991). Additionally, Western hybridisation analysis indicated that damage was also incurred on the RBP of the phage, and thus it appears the entire phage tail structure is affected by PVP, a conclusion supported by subsequent EM analysis.

A total of four biocides were selected for electron microscope imaging, and four distinct effects on phage structure were observed, all correlating with the data obtained from SDS-PAGE analysis. It appears that, in the case of Sanitizer A, the primary effect is the detachment of the tail from the phage head, resulting in the exit of phage DNA and the ensuing collapse of the capsid. Sodium hydroxide-based Sanitizer B also caused extensive damage to the head, while also resulting in the degradation of the tail structure. Thus, it appears sodium hydroxide has a broad range of targets, causing extensive damage throughout the phage structure.

In conclusion, it is apparent from the results of this study that large variations in resistance to biocidal activity are present within the 936 lactococcal phage group, the most predominant group in the dairy environment. This study corroborates the conclusion of Murphy et al. (2014), recommending sodium hydroxide as a reliable sanitizing agent for the dairy industry. We have also highlighted the inadequacy of a number of compounds due to factors such as ineffectiveness, problems associated with residues, and the formation of phage aggregates. This study has demonstrated that large variations in resistance to biocides exists between phages, and that phages resistant to biocidal activity tend to possess resistance to more than one compound. We have also demonstrated that different biocides target different areas of the phage structure. Thus, it is likely that, rather than being the result of small, precise differences between phages, resistance to biocides is due to an accumulation of features and differences between resistant and more susceptible phages, a conclusion supported by phylogenetic analysis. It is apparent that phage biocide resistance is an issue which must be carefully monitored in the dairy industry and that careful consideration must be given to the selection of biocides applied in industrial facilities. The application of composite solutions containing a mixture of biocides may mitigate this issue in combination with additional hurdles such as thermal and high-pressure treatments.

## Author contributions

DVS, SH, JMa, JMu, and AN conceived and designed experiments. MV and GL sequenced and annotated phage i0139. JN performed the mass spectrometry. HN and CF performed the electron microscopy. JMa, DVS, AN, JMu, HN, and SH reviewed, revised and edited the manuscript. All authors have read and approved the final manuscript.

## Funding

DVS the recipient of a Science Foundation Ireland (SFI) Investigator award (Ref. No. 13/IA/1953). SH is the recipient of an Irish Research Council Enterprise Partnership Scheme postgraduate scholarship. JMa is in receipt of a Technology Innovation Development Award (TIDA) (Ref. No. 14/TIDA/2287) and Starting Investigator Research Grant (SIRG) (Ref. No. 15/SIRG/3430) funded by SFI. We acknowledge the financial support from the Hercules Foundation in the framework of the project R-3986 “LC–MS@UHasselt: Linear Trap Quadrupool-Orbitrap mass spectrometer.”

### Conflict of interest statement

The authors declare that the research was conducted in the absence of any commercial or financial relationships that could be construed as a potential conflict of interest.
