# Supplementary material for: Biocidal Inactivation of Lactococcus lactis Bacteriophages: Efficacy and Targets of Commonly Used Sanitizers
Source: Front Microbiol. 2017 Feb 2;8:107. doi: 10.3389/fmicb.2017.00107 (PMC5288689; doi:10.3389/fmicb.2017.00107)
Supplement: Supplementary file 1 [file Table1.docx]

Supplementary Material

**Biocidal inactivation of *Lactococcus lactis* bacteriophages: efficacy and targets of commonly used sanitizers.**

Stephen Hayes, James Murphy, Jennifer Mahony, Gabriele Andrea Lugli, Marco Ventura, Jean-Paul Noben, Charles M.A.P. Franz, Horst Neve, Arjen Nauta, Douwe van Sinderen*

*** Correspondence:** Corresponding Author: [d.vansinderen@ucc.ie](mailto:d.vansinderen@ucc.ie)

**Supplementary Table S1**: Phage titers upon exposure to 0.08 % Benzalkonium Chloride.

| **Phage (Host)** | **Pre-Exposure** | **2 min.** | **10 min.** | **20 min.** | **30 min.** |
| --- | --- | --- | --- | --- | --- |
|  |  |  |  |  |  |
| **G (G)** | **7.09 ± 0.8** | **6.63 ± 0.1** | **6.15 ± 0.06** | **5.24 ± 0.05** | **5.19 ± 0.16** |
| A.16 (A) | 7.95 ± 0.26 | 6.28 ± 0.87 | 4.21 ± 0.44 | 3.75 ± 0.17 | 4.07 ± 0.39 |
| 19 (A) | 7.76 ± 0.15 | 5.61 ± 0.79 | 4.32 ± 0.19 | 3.86 ± 0.48 | 3.66 ± 0.51 |
| 4 (A) | 7.73 ± 0.43 | 5.8 ± 0.37 | 4.36 ± 0.45 | 3.62 ± 0.41 | 3.29 ± 0.85 |
| A1127 (A) | 7.49 ± 0.31 | 6.12 ± 0.32 | 4.39 ± 0.24 | 3.94 ± 0.13 | 3.73 ± 0.41 |
| Lj (A) | 6.97 ± 0.52 | 6.06 ± 0.19 | 5.14 ± 0.11 | 4.35 ± 0.14 | 3.93 ± 0.23 |
| C0139 (C) | 7.75 ± 0.21 | 5.37 ± 0.42 | 3.89 ± 0.68 | 3.12 ± 0.31 | 1.15 ±1.63 |
| D.18 (D) | 7.85 ± 0.14 | 5.23 ± 0.71 | 4.16 ± 1.35 | 3.2 ± 0.56 | 1.66 ± 1.5 |
| 43 (D) | 7.58 ± 0.31 | 5.8 ± 0.28 | 3.65 ± 0.34 | 3.1 ± 0.28 | 2.95 ± 0.49 |
| E.17 (E) | 7.35 ± 0.16 | 5.99 ± 0.4 | 4.74 ± 0.85 | 4.19 ± 0.84 | 3.41 ± 1.05 |
| E1127 (E) | 7.73 ± 0.2 | 5.78 ± 0.36 | 4.18 ± 0.25 | 3.75 ± 0.62 | 3.08 ± 0.42 |
| F0139 (F) | 7.55 ± 0.31 | 5.79 ± 0.23 | 4.32 ± 0.48 | 4.06 ± 0.37 | 3.58 ± 0.42 |
| F.17 (F) | 7.3 ± 0.42 | 4.65 ± 1.17 | 3.96 ± 0.28 | 3.43 ± 0.25 | 2.46 ± 0.28 |
| 4.2 (4) | 7.35 ± 0.32 | 5.78 ± 0.25 | 4.32 ± 0.34 | 3.72 ± 0.14 | 3.15 ± 0.42 |
| 5.12 (5) | 6.6 ± 0.4 | 5.28 ± 0.11 | 3.94 ± 0.2 | 3.38 ± 0.2 | 3 ± 0.83 |
| 91127 (9) | 7.62 ± 0.17 | 6.23 ± 0.35 | 5.05 ± 0.09 | 4.32 ± 0.36 | 3.61 ± 0.47 |
| 19.2 (19) | 7.31 ± 0.49 | 5.94 ± 0.1 | 3.72 ± 0.16 | 3.35 ± 0.13 | 1.39 ± 1.96 |
| 19.3 (19) | 7.38 ± 0.33 | 5.86 ± 0.1 | 4.03 ± 0.5 | 3.53 ± 0.46 | 3.14 ± 0.31 |
| 13.16 (13) | 7.73 ± 0.17 | 5.86 ± 0.19 | 3.34 ± 0.47 | 2.94 ± 0.06 | 2.58 ± 0.57 |
| 15 (13) | 7.66 ± 0.24 | 5.27 ± 0.18 | 3.42 ± 0.86 | 2.30 ± 0.07 | 2.4 ± 0.17 |
| L.18 (L) | 7.45 ± 0.2 | 5.65 ± 0.07 | 4.46 ± 0.14 | 3.58 ± 0.25 | 2.76 ± 0.45 |
| 114 (11) | 7.13 ± 0.13 | 5.35 ± 0.16 | 3.75 ± 0.33 | 2.85 ± 0.43 | 2.4 ± 0.17 |
| 129 (M) | 7.4 ± 0.13 | 5.9 ± 0.24 | 4.3 ± 0.25 | 3.69 ± 0.35 | 3.1 ± 0.3 |
| 145 (M) | 7.23 ± 0.23 | 6.42 ± 0.14 | 5.22 ± 0.19 | 4.59 ± 0.32 | 4.05 ± 0.35 |
| 109 (M) | 7.83 ± 0.1 | 6.2 ± 0.33 | 5.12 ± 0.3 | 4.47 ± 0.15 | 4.11 ± 0.24 |
| M.5 (M) | 7.06 ± 0.12 | 5.46 ± 0.06 | 3.61 ± 0.41 | 2.78 ± 0.48 | 2.62 ± 0.28 |
| 93 (M) | 7.21 ± 0.21 | 5.67 ± 0.44 | 4.62 ± 0.2 | 4.16 ± 0.03 | 3.2 ± 0.6 |
| M.16 (M) | 7.26 ± 0.21 | 5.16 ± 0.35 | 3.81 ± 0.43 | 2.9 ± 0.15 | nd |
| M1127 (M) | 7.37 ± 0.13 | 5.45 ± 0.11 | 4.27 ± 0.1 | 3.38 ± 0.68 | nd |
| 155 (M) | 7.63 ± 0.3 | 5.52 ± 0.1 | 4.06 ± 0.24 | 3.47 ± 0.29 | nd |
| L.6 (L) | 7.49 ± 0.09 | 5.21 ± 0.3 | 3.37 ± 0.38 | 2.84 ± 0.4 | nd |
| 40 (L) | 7.09 ± 0.1 | 4.79 ± 0.28 | 3.94 ± 0.06 | 3.54 ± 0.33 | nd |
| i0139 (i) | 7.75 ± 0.14 | 5.69 ± 0.11 | 3.56 ± 0.38 | 2.87 ± 0.51 | nd |
| 44 (11) | 7.5 ± 0.18 | 4.16 ± 0.55 | 3.75 ± 0.31 | 2.84 ± 0.47 | nd |
| 10.5 (10) | 7.72 ± 0.32 | 5.25 ± 0.23 | 3.95 ± 0.15 | 3.22 ± 0.23 | nd |
| 16 (9) | 6.66 ± 0.11 | 3.94 ± 0.2 | 2.42 ± 0.72 | nd | nd |
|  |  |  |  |  |  |

Phage titer is expressed in log form. nd = Not Detectable, indicating no phage survived beyond this point. **Bold** denotes resistant phages. All figures are the result of at least triplicate assays.

**Supplementary Table S2**: Phage titers after exposure to 14 % Hydrogen Peroxide.

| **Phage (Host)** | **Before Exposure** | **2 min.** | **10 min.** | **20 min.** | **30 min.** |
| --- | --- | --- | --- | --- | --- |
|  |  |  |  |  |  |
| A.16 (A) | 7.3 ± 0.2 | 6.9 ± 0.8 | 6.3 ± 0.23 | 4.46 ± 0.28 | 2.78 ± 0.64 |
| 19 (A) | 7.46 ± 0.32 | 7.18 ± 0.35 | 6.02 ± 0.15 | 4.89 ± 0.76 | 3.48 ± 0.3 |
| 4 (A) | 7.45 ± 0.28 | 6.7 ± 0.51 | 5.09 ± 0.23 | 4.16 ± 0.42 | 3.4 ± 0.45 |
| A1127 (A) | 7.42 ± 0.26 | 6.3 ± 0.36 | 5.7 ± 0.5 | 4.5 ± 0.31 | 3.3 ± 0.23 |
| Lj (A) | 7.54 ± 0.34 | 6.81 ± 0.62 | 5.61 ± 0.22 | 4.39 ± 0.31 | 3.67 ± 0.24 |
| C0139 (C) | 7.03 ± 0.13 | 6.39 ± 0.33 | 5.15 ± 0.46 | 3.51 ± 0.31 | 2.45 ± 0.42 |
| G (G) | 6.92 ± 0.41 | 6.81 ± 0.29 | 5.45 ± 0.38 | 4.51 ± 0.62 | 2.3 ± 0.23 |
| 91127 (9) | 7.51 ± 0.43 | 6.63 ± 0.67 | 6.34 ± 0.69 | 4.35 ± 0.34 | 3.54 ± 0.35 |
| 16 (9) | 7.4 ± 0.47 | 6.54 ± 0.32 | 5.97 ± 0.42 | 3.66 ± 0.68 | 2.52 ± 0.65 |
| 13.16 (13) | 7.68 ± 0.48 | 6.3 ± 0.21 | 5.85 ± 0.7 | 4.36 ± 0.28 | 2.6 ± 0.4 |
| 15 (13) | 7.28 ± 0.19 | 6.9 ± 0.68 | 6.6 ± 0.41 | 4.04 ± 0.11 | 2.6 ± 0.42 |
| 19.2 (19) | 7.64 ± 0.43 | 6.78 ± 0.6 | 5.7 ± 0.67 | 4.64 ± 0.43 | 3.53 ± 0.34 |
| 19.3 (19) | 6.82 ± 0.46 | 5.47 ± 0.29 | 4.6 ± 0.4 | 3.81 ± 0.64 | 2.3 ± 0.22 |
| 114 (11) | 7.49 ± 0.43 | 7.49 ± 0.43 | 6.52 ± 0.85 | 5.43 ± 0.6 | 3.43 ± 0.65 |
| 129 (M) | 7.37 ± 0.45 | 6.68 ± 0.62 | 5.54 ± 0.31 | 4.46 ± 0.39 | 3.44 ± 0.43 |
| 145 (M) | 7.55 ± 0.39 | 6.81 ± 0.42 | 5.76 ± 0.21 | 4.51 ± 0.54 | 2.6 ± 0.37 |
| 109 (M) | 7.56 ± 0.76 | 6.69 ± 0.37 | 5.58 ± 0.36 | 4.55 ± 0.23 | 2.6 ± 0.32 |
| M.5 (M) | 7.72 ± 0.76 | 7.13 ± 0.23 | 6.82 ± 0.63 | 3.93 ± 0.35 | 3.41 ± 0.25 |
| 93 (M) | 7.75 ± 0.56 | 7 ± 0.17 | 6.2 ± 0.16 | 4.22 ± 0.16 | 2.3 ± 0.2 |
| M1127 (M) | 7.68 ± 0.2 | 6.88 ± 0.23 | 6.41 ± 0.57 | 4.57 ± 0.35 | 2.82 ± 0.83 |
| 155 (M) | 7.38 ± 0.45 | 6.66 ± 0.68 | 6.22 ± 0.56 | 4.55 ± 0.15 | 2.16 ± 0.56 |
| M.16 (M) | 7.1 ± 0.12 | 6.37 ± 0.23 | 4.19 ± 0.15 | 2.78 ± 0.6 | nd |
| E.17 (E) | 7.11 ± 0.43 | 6.54 ± 0.76 | 5.64 ± 0.27 | 3.45 ± 0.32 | nd |
| E1127 (E) | 7.48 ± 0.32 | 6.7 ± 0.54 | 5.43 ± 0.27 | 3.2 ± 0.16 | nd |
| F.17 (F) | 7.54 ± 0.32 | 6.69 ± 0.43 | 5.4 ± 0.51 | 4.54 ± 0.72 | nd |
| L.18 (L) | 7.11 ± 0.28 | 6.98 ± 0.96 | 5.8 ± 0.62 | 3.51 ± 0.32 | nd |
| L.6 (L) | 7.71 ± 0.51 | 5.81 ± 0.64 | 5.23 ± 0.21 | 2.6 ± 0.31 | nd |
| 40 (L) | 7.08 ± 0.34 | 6.64 ± 0.52 | 4.8 ± 0.29 | 3.66 ± 0.17 | nd |
| 10.5 (10) | 7.13 ± 0.56 | 6.43 ± 0.45 | 5.37 ± 0.57 | 3.52 ± 0.54 | nd |
| 44 (11) | 7.35 ± 0.4 | 6.23 ± 0.23 | 5.99 ± 0.57 | 3.58 ± 0.25 | nd |
| D.18 (D) | 6.91 ± 0.45 | 5.64 ± 0.31 | 4.38 ± 0.25 | 3.62 ± 0.67 | nd |
| 43 (D) | 6.08 ± 0.09 | 4.38 ± 0.25 | 3.69 ± 0.59 | nd | nd |
| F0139 (F) | 6.82 ± 0.28 | 5.25 ± 0.34 | 3.29 ± 0.42 | nd | nd |
| 4.2 (4) | 7.21 ± 0.16 | 6.41 ± 0.26 | 4.24 ± 0.17 | nd | nd |
| 5.12 (5) | 7.76 ± 0.57 | 6.59 ± 0.38 | 4.37 ± 0.23 | nd | nd |
| i0139 (i) | 6.93 ± 0.54 | 5.55 ± 0.35 | 3.44 ± 0.45 | nd | nd |
|  |  |  |  |  |  |

Phage titer is expressed in log form. nd = Not Detectable, indicating no phage survived beyond this point. **Bold** denotes resistant phages. All figures are the result of at least triplicate assays.

**Supplementary Table S3**: Phage titers after exposure to 1 % Polyvinylpyrrolidone-Iodine

| **Phage (Host)** | **Before Exposure** | **2 min.** | **10 min.** | **20 min.** | **30 min.** |
| --- | --- | --- | --- | --- | --- |
|  |  |  |  |  |  |
| **G (G)** | **7.09 ± 0.13** | **7.09 ± 0.13** | **7.09 ± 0.13** | **7.09 ± 0.13** | **7.09 ± 0.13** |
| **5.12 (5)** | **7.41 ± 0.28** | **7.41 ± 0.28** | **7.41 ± 0.28** | **7.41 ± 0.28** | **7.41 ± 0.28** |
| **i0139 (i)** | **6.41 ± 0.21** | **6.3 ± 0.32** | **5.95 ± 0.49** | **5.85 ± 0.18** | **5.78 ± 0.29** |
| **F0139 (F)** | **7.69 ± 0.11** | **6.48 ± 0.27** | **5.42 ± 0.31** | **5.11 ± 0.29** | **5.04 ± 0.29** |
| **F.17 (F)** | **7.3 ± 0.23** | **6.19 ± 0.15** | **6.74 ± 0.32** | **6.6 ± 0.45** | **6.54 ± 0.25** |
| **D.18 (D)** | **6.64 ± 0.14** | **6.56 ± 0.23** | **6.54 ± 0.36** | **6.54 ± 0.24** | **6.52 ± 0.31** |
| **43 (D)** | **6.95 ± 0.32** | **6.85 ± 0.43** | **6.83 ± 0.19** | **6.81 ± 0.28** | **6.81 ± 0.21** |
| A1127 (A) | 7.33 ± 0.33 | 4.58 ± 0.34 | 3.76 ± 0.28 | 2.6 ± 0.14 | 2.6 ± 0.19 |
| 129 (M) | 7.53 ± 0.45 | 6.34 ± 0.67 | 4.45 ± 0.28 | 3.58 ± 0.52 | 3.45 ± 0.36 |
| 19 (A) | 7.76 ± 0.56 | 3.95 ± 0.9 | 3.7 ± 0.23 | 3.45 ± 0.32 | nd |
| Lj (A) | 7.45 ± 0.27 | 5.68 ± 0.73 | 3.78 ± 0.32 | 2.6 ± 0.35 | nd |
| C0139 (C) | 7.51 ± 0.27 | 4.05 ± 0.67 | 2.9 ± 0.49 | 2.3 ± 0.15 | nd |
| L.18 (L) | 7.21 ± 0.27 | 5.91 ± 0.35 | 3.53 ± 0.74 | 2.3 ± 0.28 | nd |
| E.17 (E) | 7.51 ± 0.28 | 7.02 ± 0.45 | 4.79 ± 0.32 | 2.6 ± 0.23 | nd |
| 91127 (9) | 7.46 ± 0.37 | 6.51 ± 0.09 | 4.65 ± 0.37 | 2.3 ± 0.37 | nd |
| 16 (9) | 6.96 ± 0.27 | 5.97 ± 0.32 | 4.33 ± 0.34 | 3 ± 0 | nd |
| 19.2 (19) | 7.53 ± 0.34 | 4.28 ± 0.36 | 2.78 ± 0.46 | 2.3 ± 0.43 | nd |
| 19.3 (19) | 7.56 ± 0.38 | 3.72 ± 0.23 | 3.51 ± 0.24 | 2.6 ± 0.74 | nd |
| 4 (A) | 7.55 ± 0.31 | 4.69 ± 0.32 | 3.51 ± 0.42 | nd | nd |
| 40 (L) | 7.61 ± 0.53 | 5.58 ± 0.36 | 2.51 ± 0.36 | nd | nd |
| 145 (M) | 7.87 ± 0.36 | 4.79 ± 0.23 | 3.48 ± 0.67 | nd | nd |
| M.5 (M) | 7.8 ± 0.23 | 5.48 ± 0.25 | 2.6 ± 0.32 | nd | nd |
| 93 (M) | 7.56 ± 0.17 | 4.03 ± 0.45 | 2.3 ± 0.23 | nd | nd |
| M1127 (M) | 7.7 ± 0.43 | 4.79 ± 0.46 | 3.48 ± 0.42 | nd | nd |
| 4.2 (4) | 7.11 ± 0.37 | 4.31 ± 0.4 | 3.3 ± 0.34 | nd | nd |
| 44 (11) | 7.27 ± 0.32 | 4.11 ± 0.84 | 3.3 ± 0.57 | nd | nd |
| 15 (13) | 7.52 ± 0.45 | 3.3 ± 0.32 | 2.3 ± 0.25 | nd | nd |
| A.16 (A) | 7.13 ± 0.29 | 3.64 ± 0.12 | nd | nd | nd |
| E1127 (E) | 7.43 ± 0.13 | 3.64 ± 0.12 | nd | nd | nd |
| L.6 (L) | 7.71 ± 0.12 | 3.95 ± 0.37 | nd | nd | nd |
| 109 (M) | 7.73 ± 0.45 | 4.15 ± 0.56 | nd | nd | nd |
| 155 (M) | 7.48 ± 0.05 | 3.08 ± 0.35 | nd | nd | nd |
| 10.5 (10) | 7.49 ± 0.26 | 4.23 ± 0.67 | nd | nd | nd |
| 114 (11) | 7.26 ± 0.24 | 4.05 ± 0.53 | nd | nd | nd |
| 13.16 (13) | 7.71 ± 0.24 | 4.1 ± 0.49 | nd | nd | nd |
| M.16 (M) | 7.47 ± 0.24 | nd | nd | nd | nd |
|  |  |  |  |  |  |

Phage titer is expressed in log form. nd = Not Detectable, indicating no phage survived beyond this point. **Bold** denotes resistant phages. All figures are the result of at least triplicate assays.

**Supplementary Table S4**: Phage titers after exposure to 0.08 % Sanitiser A.

| **Phage (Host)** | **Before Exposure** | **2 min.** | **10 min.** | **20 min.** | **30 min.** |
| --- | --- | --- | --- | --- | --- |
|  |  |  |  |  |  |
| **E1127 (E)** | **7.43 ± 0.27** | **6.94 ± 0.43** | **6.6 ± 0.42** | **6.56 ± 0.21** | **6.45 ± 0.35** |
| **G (G)** | **7.29 ± 0.07** | **6.65 ± 0.24** | **6.04 ± 0.21** | **5.95 ± 0.19** | **5.21 ± 0.31** |
| **5.12 (5)** | **7.41 ± 0.25** | **6.39 ± 0.41** | **5.56 ± 0.43** | **5.11 ± 0.74** | **5.01 ± 0.29** |
| **43 (D)** | **6.85 ± 0.17** | **6.23 ± 0.47** | **5.27 ± 0.23** | **4.49 ± 0.32** | **4.23 ± 0.72** |
| A.16 (A) | 7.13 ± 0.08 | 5.46 ± 0.42 | 4.26 ± 0.34 | 3.78 ± 0.51 | 3.26 ± 0.37 |
| 19 (A) | 7.77 ± 0.21 | 6.39 ± 0.29 | 5.13 ± 0.23 | 4.44 ± 0.48 | 4.31 ± 0.29 |
| 4 (A) | 7.58 ± 0.14 | 5.63 ± 0.78 | 4.89 ± 0.32 | 4.63 ± 0.09 | 3.46 ± 0.28 |
| A1127 (A) | 7.33 ± 0.13 | 6.31 ± 0.43 | 5.06 ± 0.79 | 4.13 ± 0.05 | 3.76 ± 0.15 |
| Lj (A) | 7.37 ± 0.15 | 5.67 ± 0.23 | 4.52 ± 0.37 | 3.99 ± 0.09 | 3.54 ± 0.39 |
| E.17 (E) | 7.51 ± 0.09 | 5.26 ± 0.23 | 3.68 ± 0.23 | 3.15 ± 0.37 | 2.3 ± 0.32 |
| L.18 (L) | 7.21 ± 0.19 | 4.52 ± 0.27 | 4.51 ± 0.27 | 3.62 ± 0.23 | 2.9 ± 0.36 |
| 145 (M) | 7.8 ± 0.16 | 5.54 ± 0.37 | 4.89 ± 0.32 | 3.53 ± 0.25 | 2.6 ± 0.08 |
| 93 (M) | 7.88 ± 0.19 | 5.95 ± 0.4 | 4.64 ± 0.26 | 4.09 ± 0.37 | 3.34 ± 0.47 |
| 155 (M) | 7.61 ± 0.23 | 5.45 ± 0.32 | 4.53 ± 0.34 | 2.97 ± 0.2 | 2.6 ± 0.21 |
| 91127 (9) | 7.37 ± 0.11 | 6.62 ± 0.27 | 5.32 ± 0.23 | 4.51 ± 0.67 | 3.55 ± 0.32 |
| 16 (9) | 7.24 ± 0.25 | 6.41 ± 0.32 | 5.37 ± 0.34 | 4.89 ± 0.32 | 3.43 ± 0.38 |
| 114 (11) | 7.26 ± 0.312 | 3.87 ± 0.43 | 3.08 ± 0.08 | 2.6 ± 0.05 | 2.6 ± 0.12 |
| 44 (11) | 7.27 ± 0.31 | 4.34 ± 0.21 | 3.3 ± 0.39 | 2.9 ± 0.49 | 2.3 ± 0.23 |
| 15 (13) | 7.52 ± 0.31 | 4.85 ± 0.47 | 3.97 ± 0.24 | 3.34 ± 0.43 | 2.9 ± 0.08 |
| 19.3 (19) | 7.56 ± 0.11 | 5.92 ± 0.27 | 3.97 ± 0.3 | 3.72 ± 0.32 | 2.3 ± 0.47 |
| C0139 (C) | 7.65 ± 0.12 | 4.75 ± 0.27 | 3.51 ± 0.23 | 2.6 ± 0.23 | nd |
| D.18 (D) | 6.15 ± 0.24 | 4.66 ± 0.34 | 3.26 ± 0.25 | 2.78 ± 0.56 | nd |
| F0139 (F) | 7.09 ± 0.19 | 3.3 ± 0.23 | 3.08 ± 0.37 | 2.3 ± 0.26 | nd |
| F.17 (F) | 7 ± 0.24 | 4.52 ± 0.37 | 2.6 ± 0.4 | 2.3 ± 0.09 | nd |
| L.6 (L) | 7.71 ± 0.22 | 3.9 ± 0.37 | 2.78 ± 0.12 | 2.3 ± 0.7 | nd |
| 40 (L) | 7.53 ± 0.08 | 4.64 ± 0.21 | 3.47 ± 0.3 | 2.6 ± 0.29 | nd |
| 129 (M) | 7.74 ± 0.14 | 5.53 ± 0.21 | 3.88 ± 0.27 | 2.64 ± 0.31 | nd |
| 109 (M) | 7.75 ± 0.24 | 5.68 ± 0.27 | 4.53 ± 0.21 | 3.68 ± 0.72 | nd |
| M.5 (M) | 7.8 ± 0.19 | 5.25 ± 0.24 | 3.15 ± 0.14 | 2.6 ± 0.43 | nd |
| M.16 (M) | 7.47 ± 0.1 | 4.78 ± 0.14 | 4.76 ± 0.32 | 3.2 ± 0.12 | nd |
| M1127 (M) | 7.73 ± 0.23 | 4.98 ± 0.36 | 3.59 ± 0.34 | 3.38 ± 0.6 | nd |
| 13.16 (13) | 7.71 ± 0.23 | 5.53 ± 0.54 | 3.48 ± 0.42 | 3.26 ± 0.2 | nd |
| 19.2 (19) | 7.53 ± 0.09 | 6.07 ± 0.72 | 3.38 ± 0.34 | 2.3 ± 0.23 | nd |
| 10.5 (10) | 7.25 ± 0.32 | 4.51 ± 0.89 | 3 ± 0.2 | nd | nd |
| 4.2 (4) | 7.53 ± 0.2 | 3.08 ± 0.67 | nd | nd | nd |
| i0139 (i) | 6.41 ± 0.34 | 4.4 ± 0.13 | nd | nd | nd |
|  |  |  |  |  |  |

Phage titer is expressed in log form. nd = Not Detectable, indicating no phage survived beyond this point. **Bold** denotes resistant phages. All figures are the result of at least triplicate assays.

**Supplementary Table S5**: Phage titers after exposure to 0.1 % Sanitiser B.

| **Phage (Host)** | **Before Exposure** | **2 min.** | **10 min.** | **20 min.** | **30 min.** |
| --- | --- | --- | --- | --- | --- |
|  |  |  |  |  |  |
| **G (G)** | **7.64 ± 0.34** | **6.65 ± 0.32** | **5.75 ± 0.19** | **5.38 ± 0.17** | **5.26 ± 0.32** |
| **i0139 (i)** | **7.24 ± 0.16** | **7.12 ± 0.43** | **7.05 ± 0.47** | **6.9 ± 0.54** | **6.88 ± 0.23** |
| **F0139 (F)** | **6.41 ± 0.3** | **6.38 ± 0.42** | **6.34 ± 0.15** | **6.34 ± 0.37** | **6.3 ± 0.38** |
| **43 (D)** | **6.2 ± 0.21** | **6.15 ± 0.29** | **6.1 ± 0.24** | **5.95 ± 0.52** | **5.64 ± 0.46** |
| C0139 (C) | 7.33 ± 0.27 | 4.13 ± 0.34 | 3.45 ± 0.67 | 3.72 ± 0.26 | 3.26 ± 0.53 |
| F.17 (F) | 7.15 ± 0.31 | 4.93 ± 0.71 | 2.99 ± 0.38 | 2.6 ± 0.23 | 2.3 ± 0.42 |
| L.18 (L) | 7.18 ± 0.32 | 3.51 ± 0.32 | 3.79 ± 0.36 | 2.9 ± 0.24 | 2.78 ± 0.38 |
| 10.5 (10) | 7.49 ± 0.35 | 6.64 ± 0.54 | 4.71 ± 0.43 | 3.76 ± 0.37 | 2.78 ± 0.32 |
| 114 (11) | 7.47 ± 0.15 | 5.64 ± 0.37 | 4.41 ± 0.73 | 3.9 ± 0.34 | 3.85 ± 0.23 |
| 44 (11) | 7.57 ± 0.45 | 4.43 ± 0.39 | 3.96 ± 0.43 | 2.08 ± 0.05 | 2.08 ± 0.05 |
| D.18 (D) | 7.08 ± 0.2 | 5.53 ± 0.38 | 3.86 ± 0.36 | 2.6 ± 0.43 | nd |
| E1127 (E) | 7.38 ± 0.09 | 4.83 ± 0.12 | 3.2 ± 0.51 | 2.6 ± 0.29 | nd |
| 109 (M) | 7.73 ± 0.29 | 5.33 ± 0.34 | 3.72 ± 0.47 | 2.6 ± 0.34 | nd |
| 19.3 (19) | 7.68 ± 0.13 | 4.11 ± 0.42 | 3.6 ± 0.21 | 3.34 ± 0.29 | nd |
| A.16 (A) | 7.72 ± 0.15 | 4.62 ± 0.52 | 2.3 ± 0.12 | nd | nd |
| 19 (A) | 7.43 ± 0.15 | 4.62 ± 0.52 | 2.3 ± 0.12 | nd | nd |
| A1127 (A) | 7.47 ± 0.27 | 2.48 ± 0.3 | 2.3 ± 0.21 | nd | nd |
| 145 (M) | 7.53 ± 0.05 | 5.74 ± 0.75 | 3.53 ± 0.31 | nd | nd |
| M1127 (M) | 7.81 ± 0.23 | 5.99 ± 0.64 | 3.63 ± 0.25 | nd | nd |
| 91127 (9) | 7.6 ± 0.64 | 4.03 ± 0.43 | 3.2 ± 0.23 | nd | nd |
| 15 (13) | 7.24 ± 0.62 | 3.48 ± 0.43 | 2.6 ± 0.45 | nd | nd |
| 19.2 (19) | 7.71 ± 0.37 | 3.3 ± 0.23 | 2.78 ± 0.75 | nd | nd |
| 4 (A) | 7.11 ± 0.43 | 3.75 ± 0.25 | nd | nd | nd |
| L.6 (L) | 7.76 ± 0.34 | 2.9 ± 0.84 | nd | nd | nd |
| 40 (L) | 7.06 ± 0.34 | 2.6 ± 0.23 | nd | nd | nd |
| 129 (M) | 7.86 ± 0.23 | 4.63 ± 0.28 | nd | nd | nd |
| M.5 (M) | 7.41 ± 0.15 | 3.54 ± 0.73 | nd | nd | nd |
| M.16 (M) | 7.27 ± 0.38 | 2.3 ± 0.64 | nd | nd | nd |
| 4.2 (4) | 7.27 ± 0.43 | 3.15 ± 0.21 | nd | nd | nd |
| 16 (9) | 7.1 ± 0.54 | 2.6 ± 0.37 | nd | nd | nd |
| 13.16 (13) | 7.59 ± 0.43 | 3.78 ± 0.34 | nd | nd | nd |
| Lj (A) | 7.06 ± 0.25 | nd | nd | nd | nd |
| E.17 (E) | 7.47 ± 0.2 | nd | nd | nd | nd |
| 93 (M) | 7.77 ± 0.2 | nd | nd | nd | nd |
| 155 (M) | 7.59 ± 0.12 | nd | nd | nd | nd |
| 5.12 (5) | 7.76 ± 0.14 | nd | nd | nd | nd |
|  |  |  |  |  |  |

Phage titer is expressed in log form. nd = Not Detectable, indicating no phage survived beyond this point. **Bold** denotes resistant phages. All figures are the result of at least triplicate assays.

**Supplementary Table S6**: Phage titers after exposure to 0.12 % Sanitiser C.

| **Phage (Host)** | **Before Exposure** | **2 min.** | **10 min.** | **20 min.** | **30 min.** |
| --- | --- | --- | --- | --- | --- |
|  |  |  |  |  |  |
| **G (G)** | **7.64 ± 0.09** | **7.64 ± 0.14** | **7.63 ± 0.21** | **7.62 ± 0.32** | **7.61 ± 0.24** |
| **5.12 (5)** | **7.76 ± 0.24** | **7.62 ± 0.37** | **7.6 ± 0.53** | **6.95 ± 0.21** | **6.7 ± 0.29** |
| **43 (D)** | **6.68 ± 0.11** | **6.61 ± 0.31** | **6.58 ± 0.14** | **6.54 ± 0.43** | **6.51 ± 0.23** |
| **F0139 (F)** | **6.41 ± 0.14** | **6.3 ± 0.34** | **6.25 ± 0.32** | **6.08 ± 0.12** | **5.98 ± 0.38** |
| **i0139 (i)** | **6.48 ± 0.21** | **6.3 ± 0.32** | **6 ± 0.14** | **5.95 ± 0.25** | **5.9 ± 0.13** |
| 19 (A) | 7.59 ± 0.24 | 5.41 ± 0.49 | 4,81 ± 0.76 | 3.45 ± 0.32 | 2.3 ± 0.26 |
| A1127 (A) | 7.47 ± 0.43 | 3.98 ± 0.32 | 3.38 ± 0.48 | 2.78 ± 0.32 | 2.3 ± 0.12 |
| E.17 (E) | 7.47 ± 0.32 | 5.54 ± 0.34 | 3.2 ± 0.32 | 3.15 ± 0.47 | 2.78 ± 0.42 |
| E1127 (E) | 7.38 ± 0.23 | 4.56 ± 0.42 | 4.26 ± 0.26 | 3.62 ± 0.31 | 3.47 ± 0.78 |
| L.18 (L) | 7.18 ± 0.19 | 4.12 ± 0.32 | 3.48 ± 0.23 | 3.2 ± 0.72 | 2.6 ± 0.45 |
| L.6 (L) | 7.76 ± 0.13 | 5.71 ± 0.12 | 4.33 ± 0.32 | 4.18 ± 0.43 | 3.98 ± 0.82 |
| 40 (L) | 7.06 ± 0.32 | 3.9 ± 0.43 | 3.85 ± 0.23 | 3.41 ± 0.47 | 2.6 ± 0.37 |
| 129 (M) | 7.75 ± 0.2 | 4.62 ± 0.27 | 3.41 ± 0.32 | 2.9 ± 0.21 | 2.3 ± 0.23 |
| 145 (M) | 7.54 ± 0.09 | 5.72 ± 0.19 | 4.75 ± 0.34 | 4.45 ± 0.1 | 3.3 ± 0.32 |
| 109 (M) | 7.64 ± 0.19 | 4.3 ± 0.41 | 3.9 ± 0.73 | 3.46 ± 0.23 | 2.78 ± 032 |
| M.5 (M) | 7.7 ± 0.23 | 4.63 ± 0.47 | 3.2 ± 0.21 | 3.15 ± 0.23 | 2.3 ± 0.32 |
| 93 (M) | 7.77 ± 0.23 | 4.88 ± 0.18 | 4.82 ± 0.23 | 4.23 ± 0.42 | 4.11 ± 0.21 |
| M.16 (M) | 7.27 ± 0.27 | 4.21 ± 0.23 | 4.09 ± 0.49 | 4.09 ± 0.48 | 3.26 ± 0.68 |
| M1127 (M) | 7.78 ± 0.28 | 5.6 ± 0.17 | 4.53 ± 0.28 | 4.45 ± 0.32 | 4.3 ± 0.08 |
| 155 (M) | 7.59 ± 0.3 | 4.4 ± 0.74 | 3.94 ± 0.37 | 3.6 ± 0.23 | 3.58 ± 0.65 |
| 91127 (9) | 7.6 ± 0.31 | 4.85 ± 0.32 | 4.56 ± 0.73 | 4.06 ± 0.08 | 3.48 ± 0.31 |
| 16 (9) | 7.1 ± 0.21 | 3.75 ± 0.43 | 3.3 ± 0.28 | 3.38 ± 0.45 | 3.08 ± 0.27 |
| 10.5 (10) | 7.49 ± 0.16 | 5.89 ± 0.23 | 4.57 ± 0.43 | 2.78 ± 0.34 | 2.3 ± 0.46 |
| 44 (11) | 7.57 ± 0.21 | 3.9 ± 0.32 | 2.6 ± 0.23 | 2.6 ± 0.23 | 2.3 ± 0.13 |
| 15 (13) | 7.24 ± 0.15 | 4.49 ± 0.23 | 4 ± 0.78 | 3.45 ± 0.2 | 3.38 ± 0.43 |
| 19.2 (19) | 7.46 ± 0.2 | 4.31 ± 0.27 | 4.3 ± 0.63 | 4.19 ± 0.43 | 3.97 ± 0.42 |
| 19.3 (19) | 6.68 ± 0.13 | 3.3 ± 0.73 | 3.3 ± 0.27 | 3 ± 0.32 | 2.78 ± 0.32 |
| A.16 (A) | 7.63 ± 0.09 | 5.39 ± 0.43 | 3.44 ± 0.29 | 2.37 ± 0.18 | nd |
| 4 (A) | 7.11 ± 0.28 | 6 ± 0.26 | 3 ± 0.35 | 2.6 ± 0.23 | nd |
| Lj (A) | 7.06 ± 0.22 | 4.5 ± 0.62 | 3.08 ± 0.12 | 2.78 ± 0.32 | nd |
| D.18 (D) | 7.08 ± 0.3 | 4.53 ± 0.41 | 2.45 ± 0.12 | 2.3 ± 0.23 | nd |
| F.17 (F) | 7.28 ± 0.32 | 4.45 ± 0.32 | 3.51 ± 0.87 | 2.9 ± 0.31 | nd |
| 114 (11) | 7.53 ± 0.24 | 3.62 ± 0.26 | 2.9 ± 0.74 | nd | nd |
| C0139 (C) | 7.33 ± 0.14 | 3.87 ± 0.81 | nd | nd | nd |
| 4.2 (4) | 7.27 ± 0.14 | 4.25 ± 0.52 | nd | nd | nd |
| 13.16 (13) | 7.59 ± 0.21 | 3.34 ± 0.43 | nd | nd | nd |
|  |  |  |  |  |  |

Phage titer is expressed in log form. nd = Not Detectable, indicating no phage survived beyond this point. **Bold** denotes resistant phages. All figures are the result of at least triplicate assays.

>Phi93_15 Phi93_15 Major tail protein

MKLDYNSREIFFGNEALIVADMAKGSNGKLEFTNHKIVTGLVSVGSMEDQAETNSYPADD

VPDHGVKKGATLLQGEMVFIQTDQALKEDILGQQRTANGLGWSTTGDWKTKCVQYLIKGR

KRDKVTGEFIDGYRVVVYPKLKPTAEPTKESETDSVDGVDPIQWTLAVQATESDIYLNNG

KNVATIEYEIWGEQAKDFAKKMESGLFIMQPDTVLAGAVTLVAPVIPNVTTTRRGGNDGT

IVVPDTLKDSNGGTVKVTSVIKDANGKVETNGHLAPGVHLVTFSADGYQDVNSGVSVTDH

P

**Supplementary Figure S1:** Amino acid sequence of the Major Tail Protein (MTP) of phage 93, with peptide reads obtained from mass spec analysis of protein bands obtained after PVP exposure underlined. Single underline denotes those reads obtained from the smallest band of 11 kDa. Double underline denotes those reads obtained from the two larger bands of 23 kDa and 46 kDa. From this, it was possible to narrow the area targeted by PVP to the thirteen amino acid residues highlighted in red.


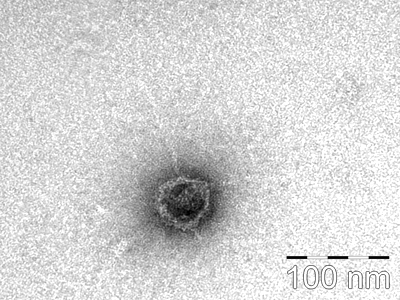

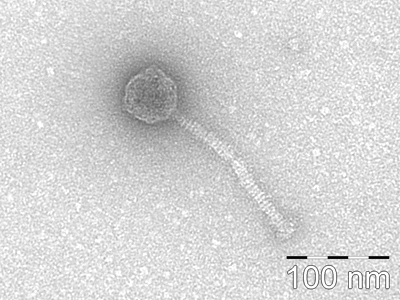

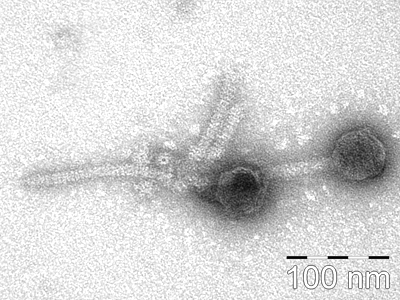

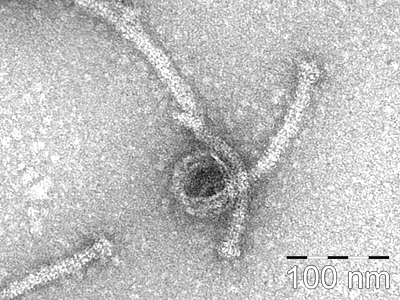

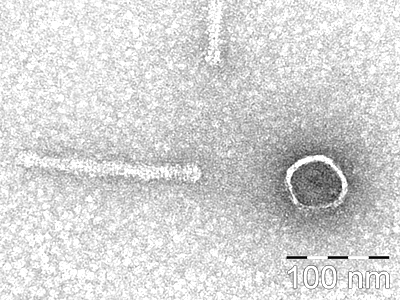

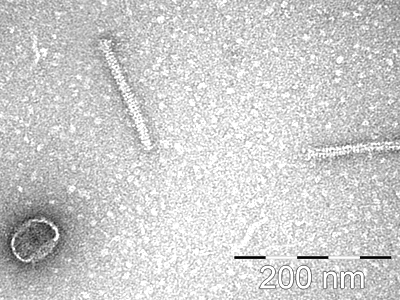

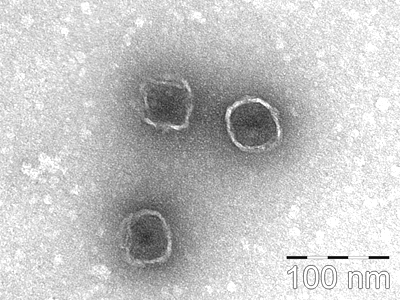

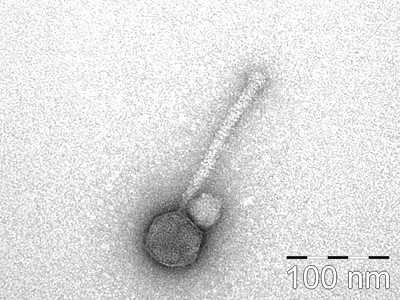

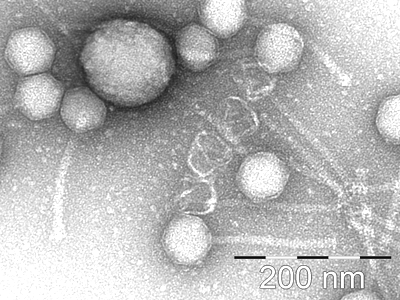


**A.**

**C.**

**D.**

**F.**

**E.**

**I.**

**H.**

**G.**

**B.**

**Supplementary Figure S2**: Additional images of the effects of biocide exposure on the structure of phage 93. A: 3 % PVP. B: 1 % PVP with break-point of tail highlighted in red. C: 1 % PVP with possible detached baseplates highlighted in red. D: 0.5 % Sanitiser A. E: 1 % Sanitiser A. F: 0.5 % Sanitiser B. G: 0.1 % Sanitiser B. H: 1 % Sanitiser E. I: 3 % Sanitiser E.
